# Draft Genome Sequence of *Pseudomonas* sp. Strain LFM046, a Producer of Medium-Chain-Length Polyhydroxyalkanoate

**DOI:** 10.1128/genomeA.00966-15

**Published:** 2015-08-20

**Authors:** Juliana Cardinali-Rezende, Paulo Moises Raduan Alexandrino, Rafael Augusto Theodoro Pereira de Souza Nahat, Débora Parrine Vieira Sant’Ana, Luiziana Ferreira Silva, José Gregório Cabrera Gomez, Marilda Keico Taciro

**Affiliations:** a Institute of Biomedical Sciences, University of São Paulo, São Paulo, Brazil; b Department of Computer Science, Institute of Mathematics and Statistics, University of São Paulo, São Paulo, Brazil

## Abstract

*Pseudomonas* sp. LFM046 is a medium-chain-length polyhydroxyalkanoate (PHA_MCL_) producer capable of using various carbon sources (carbohydrates, organic acids, and vegetable oils) and was first isolated from sugarcane cultivation soil in Brazil. The genome sequence was found to be 5.97 Mb long with a G+C content of 66%.

## GENOME ANNOUNCEMENT

*Pseudomonas* sp. LFM046 was isolated from sugarcane soil in Brazil as a polyhydroxyalkanoate (PHA_MCL_) producer (1), presenting outstanding performance in producing such polymers from carbohydrates (2, 3) or plant oils (4) when compared to the reference strain *Pseudomonas putida* KT2440. Previous flux analysis of central metabolism using [U-^13^C]glucose revealed an atypical behavior (5), suggesting that *Pseudomonas* sp. LFM046 could be a new platform for the synthesis of other biobased products.

The strain’s whole-genome DNA was extracted using a DNeasy blood and tissue kit (Qiagen, Valencia, CA, USA) and quantified using Qubit 2.0 fluorometer (Life Technology, USA). We obtained 550-bp DNA fragments using Covaris S2 (Covaris, Inc.), subsequently visualized on agarose gel electrophoresis, and quantified using a Qubit 2.0 fluorometer. A paired-end sequencing library was constructed using a True Seq DNA PCR Free LT sample preparation kit (Illumina, San Diego, CA, USA). Library insert size was verified using a 2100 Bioanalyzer (Agilent Technologies, USA) and quantified by real-time PCR (qPCR) using a library quantification kit for the Illumina genome analyzer (KAPA Biosystems, Massachusetts, USA). The sequencing library was prepared according to the manufacturer’s protocols and sequenced on an Illumina MiSeq sequencer (Illumina, San Diego, CA, USA).

A total of 3,700,436 reads (forward and reverse) were generated, decreasing to 3,032,982 reads after quality control using Galaxy software (https://usegalaxy.org/). *De novo* genome assembly was performed using Velvet Assembly (6) (considering k-mer = 97, cov k-mer = 22, and reads > 200 bp), and contig annotation was carried out using the Rapid Annotation Subsystem Technology (RAST) server (7). A draft genome composed of 34 contigs was obtained with a maximum contig size of 1,038,386 bp and an *N*_50_ contig of 640,128 bp. The genome was found to be 5,970,318 bp long with 60-fold coverage and a G+C content of 66%; 5,440 coding sequences and 75 RNAs were annotated. Four hundred forty-five genes were related to carbohydrate metabolism, including 204 genes involved in carbohydrate central metabolism, organized as peripheral glucose catabolism (8 genes); methylglyoxal metabolism (43 genes); pyruvate metabolism II: acetyl-CoA, acetogenesis from pyruvate (37 genes); pyruvate-alanine-serine interconversions (16 genes); glyoxylate bypass (8 genes); glycolysis and gluconeogenesis (13 genes); Entner-Doudoroff pathway (16 genes); dehydrogenase complexes (15 genes); TCA cycle (18 genes); pyruvate metabolism I: anaplerotic reactions, PEP (12 genes); pentose phosphate pathway (10 genes); and glycolate-glyoxylate interconversions (8 genes).

The presence of genes from β-oxidation and biosynthesis of fatty acids were also investigated since they contribute precursors to PHA_MCL_ biosynthesis from plant oils and carbohydrates, respectively. Thirty-six genes from β-oxidation and 46 from fatty acids biosynthesis were identified. The fine prediction and annotation of genes involved in PHA_MCL_ biosynthesis, carbohydrates, and fatty acid catabolism are in progress to build up a genomic scale metabolic network. To improve PHA_MCL_ production and evaluate the potential of the strain to generate new biotechnological compounds, *in silico* experiments considering the resulting network are being conducted.

### Nucleotide sequence accession numbers.

The contig sequences generated from the whole-genome sequence of *Pseudomonas* sp. LFM046 were deposited in the DDBJ/EMBL/GenBank database under the accession number JYKO00000000. The version described in this paper is the first version, JYKO01000000.
